# The hope, hype and obstacles surrounding cell therapy

**DOI:** 10.1111/jcmm.18359

**Published:** 2024-05-21

**Authors:** Cezary Tręda, Aneta Włodarczyk, Piotr Rieske

**Affiliations:** ^1^ Department of Tumor Biology Medical University of Lodz Lodz Poland

**Keywords:** CAR‐T, cell therapy, stem cells, synthetic biology

## Abstract

Cell therapy offers hope, but it also presents challenges, most particularly the limited ability of human organs and tissues to regenerate. Since many diseases are associated with irreversible pathophysiological or traumatic changes, stem cells and their derivatives are unable to secure healing. Although regenerative medicine offers chances for improvements in many diseases, such as type one diabetes and Parkinson's disease, it cannot eliminate the primary cause of many of them. While successes can be expected for diseases such as sickle cell disease, this is not the case for hereditary diseases with varied mutation types or for ciliopathies, which start in embryogenesis. In this complicated medical environment, synthetic biology offers some solutions, but their implementation will take many years. Still, positive examples such as CAR‐T therapy offer hope.

## 1 COMPARING INDUCED PLURIPOTENT STEM CELLS WITH MULTIPOTENT CELLS

**FIGURE 1:**
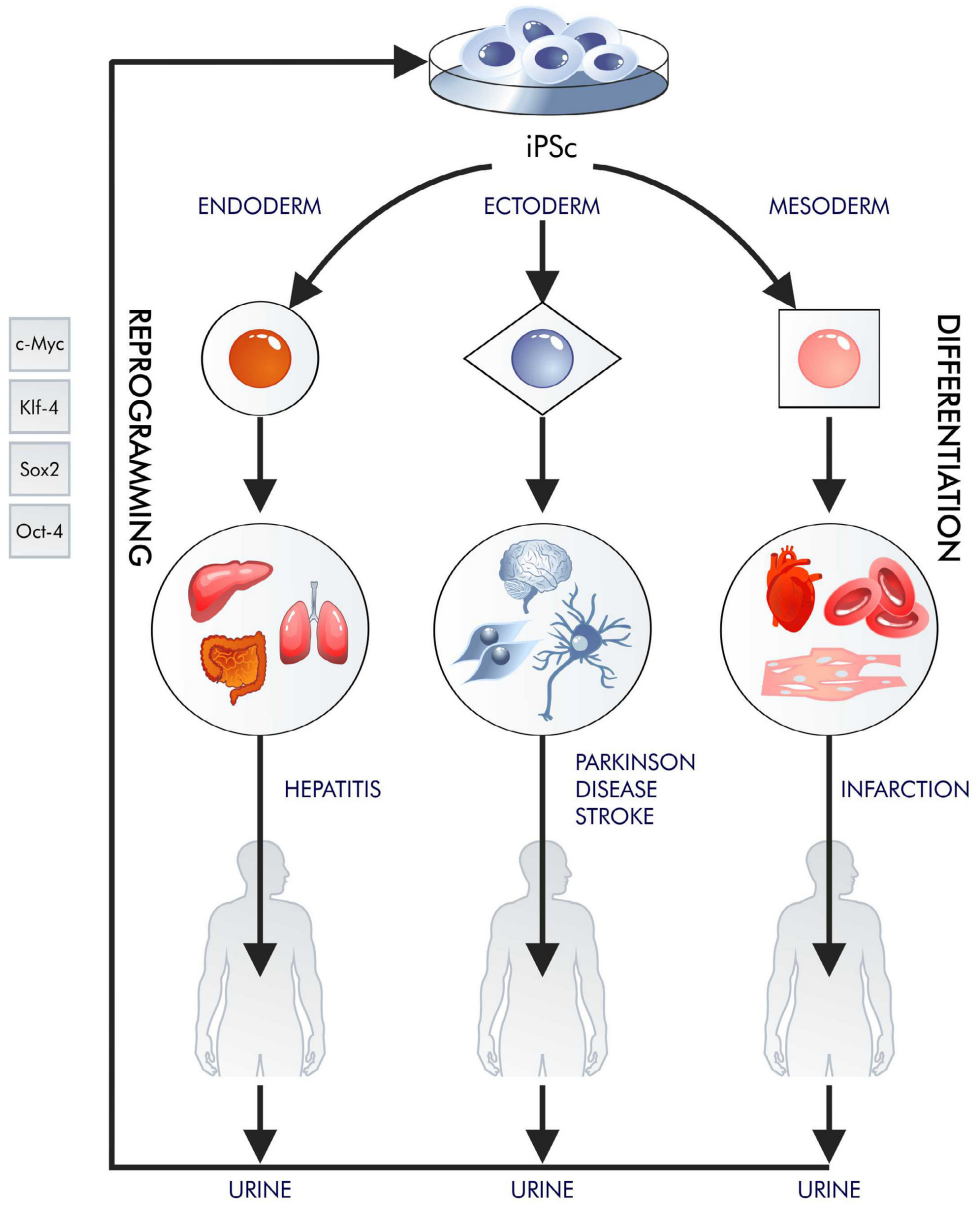
Reprogramming to iPSC is a phenomenon that has been known for several years. At the moment, not only fibroblasts can be reprogrammed, but also, for example, urine cells. Those cells can be collected from patients with many diseases and reprogrammed into iPSCs, which are differentiated into the cells required by patients.

Most of the so‐called emerging technologies, such as various branches of cell therapy, develop according to the Gartner curve; they evolve slowly and often encounter similar problems as other emerging technologies. In these cases, cell therapy is beset by fundamental biological problems related to our limited ability to regenerate.^1^ However, it is possible to obtain more pluripotent cells in vitro than tissues provide multipotent in vivo. Pluripotent stem cells (PSCs) can be obtained from most mature cells through reprogramming; these can differentiate into cell types needed by patients and multiply for an infinite amount of time (Figure 1). Shinya Yamanaka and Kazutoshi Takahashi (2006)^2^ selected four proteins (OCT4, SOX2, KLF4 and MYC (OSKM)) through targeted selection. Their discovery eliminated the necessity for cell nucleus transfer into an egg cell by enabling the amplification of the four transcription factors within the nucleus of a mature cell.

From an ethical perspective, it is important that pluripotency is achieved rather than totipotency: totipotent stem cells can give rise to an entire organism, whereas pluripotent stem cells can give rise to all types of an adult organism but not whole organisms since iPSC cannot differentiate to structures such as yolk sac or placenta. Generating whole organisms raises ethical and legal issues.

While the changes that occur step‐by‐step during reprogramming are unknown, a continuum of changes slowly reveals itself. Steps such as genomic OCT‐4 SOX‐2 restoration, NANOG expression and mesenchymal‐to‐epithelial transition have been explained quite well. However, the entire process of reprogramming appears to be stochastic and is based on many trajectories.^3^ Only a few colonies of early induced PS cells (iPSCs) are generated from thousands of cells that express all four transcription factors required for reprograming. Therefore, it can be extremely difficult to precisely define this process.^4^

Comparing the time‐reprogramming processes of human cells to moving traditional clock hands backwards is an inaccurate analogy. The discoverers of this process acknowledged this distinction from the outset, opting to term it ‘reprogramming’. It resembles more of a reset than a mechanical reversal of the sequence of events that occurred at the time of differentiation — a turning back of the clock.^5^

However, it is worth noting that in female cells undergoing reprogramming, X chromosomes are only likely to undergo lyonization during advanced reprogramming, particularly when iPSC transform into naive iPSC.^6^ This transition from iPSC to naive iPSC has demonstrated the potential for a deeper epigenetic genome reset. Epigenetic changes play a crucial role during differentiation by silencing pluripotency (stemness) and enhancing the expression of mature cell genes. They are also important in the regulation of transcription factors involved in differentiation, which can be activated or inactivated by, inter alia, DNA methylation and histone modifications.

When reprogramming of mature cells occurs, OSKM transgenes gradually cause changes in the epigenetic status and expression of their endogenous replacements. These changes are associated with a cascade of epigenetic processes. For example, OCT‐4 binds to regulatory elements of gene clusters, including those encoding miRNA (miR302, miR124 and miR135).^7^

Chromatin and active gene promoters are characterized by H3K27ac and H3K9ac (characteristic histone modifications — histone code), whereas silenced genes are characterized by H3K9me3 and H3K27me3. The DNA of inactive genes shows methylated cytosine. Interestingly, exogenous (but not endogenous) OCT‐4 can be replaced by DNA demethylase Tet1, but in general, the OSKM transcription factors eliminate repressive methylation in the promoter regions of stemness genes while simultaneously enhancing cell lineage‐specific genes. The levels of endogenous proteins such as Oct4, SOX2 and NANOG are gradually increasing during reprogramming, and their gene promoters are epigenetically changing to allow access to enhancers.^8^, ^9^, ^10^, ^11^

Cells can be taken for cell therapy from foetuses, adult donors, embryonic stem cells (ESCs), and multipotent and induced pluripotent stem cells (MSCs and iPSCs, respectively). Fetal cells are not commonly used due to ethical concerns, while ESCs are controversial due to their embryonic origins. Adult donor cells are a viable option, but they have limited differentiation potential. Each cell type has a different ability to differentiate, risk of creating teratomas and degree of legal regulation. The cell types are compared in Table 1.

**TABLE 1:** Comparative analysis of stem cell types for future therapies.

	Cell type			
	iPSC	ESC	Fetal	MSC
Autologous transplant	Yes	No. Cloning is banned in most countries	No	Yes
Differentiation potential	Versatile	Versatile	Multipotent	Multipotent
Teratomas	Small risks, eliminated increasingly effectively	Small risks, eliminated increasingly effectively	No	No
Legal restrictions	Minor	Yes	Yes	Minor
Genome editing	Yes	No. genome editing is restricted in most countries	No	Minor use
Division limit	No	No	Yes	Yes

iPS cells are, of course, essential and offer promise when it comes to therapeutic options. However, while properly cultured iPSCs can multiply in vitro without any restrictions, yielding the unlimited number of required differentiated cells, this may lead to serious safety concerns in vivo, insofar as uncontrolled proliferation and differentiation of iPSCs could lead to the formation of teratomas. Those concerns were addressed by implementing two primary methodologies: in vivo tumorigenicity assays^12^ and genetic integrity validation.^13^ In vivo assays involve implanting cells into animal models to assess tumour formation, while genetic integrity validation focuses on analysing genomic stability and the presence of mutations. Tumorigenic potential can be mitigated by strategies such as optimizing reprogramming methods, cell purification and utilizing exogenous DNA‐free vectors, such as episomes.^14^, ^15^ Clinical trial results to date indicate that the current methodology for iPSC preparation for transplantation proves to be safe. In two completed clinical trials,^16^, ^17^ a copy number change was detected in the iPSC‐derived retinal pigment epithelium in one case, but it did not lead to the formation of a teratoma. Additionally, researchers are constantly improving the process of iPSC reprogramming to prevent the cells from reverting back to their original state after they have been differentiated.^14^ They are also working on methods to remove any remaining iPSCs from the transplant preparations.^17^ Allogeneic or autologous MSCs do not have the ability to form teratomas, which are a potential concern with iPSCs.^18^ However, the pluripotency of iPSCs exceeds MSCs' significantly. This is why it is important to continue studying and developing transplantations using iPSCs, even though the process of reprogramming is quite time‐consuming, taking around 2–3 months to complete. This can pose a serious problem in cases where patients require such a cellular drug immediately.^19^ Furthermore, iPSC differentiation can be performed with varying degrees of efficiency and effectiveness. While it is relatively easy to obtain neural stem cells^20^ and then neurons^21^ or cardiomyocytes,^22^ it takes much longer to obtain astrocytes.^23^ It can also be very difficult to obtain a single required fraction or type of mature cell, as differentiating cells support each other; for example, astrocytes support neurons.^24^ It is even more difficult to obtain certain cell types, such as those that produce or secrete insulin in a glucose‐dependent manner,^25^ or hepatocytes. It is easier to produce cells that secrete insulin, similar to fetal‐insulin‐producing cells, which do not respond to changes in glucose concentration^26^; however, cells that secrete insulin in a chaotic manner can do more harm than good to a diabetic patient.^27^

It is also possible for iPSCs to transform into teratomas in vivo. Research is underway to try to eliminate this problem,^15^ with attempts being made to use multipotent cells such as mesenchymal stem cells and allogeneic cells. Attempts have also been made to reprogram mature cells into multipotent cells, such as NSCs (neural stem cells), but without much success, possibly due to their relatively rapid senescence, which prevents the formation of other single iMSC‐reprogrammed (induced multipotent stem cell) colonies arising from such single iPSC.^28^ It is also possible that obtaining iPSCs depends on a certain epigenetic reset of the entire chromatin, which is not possible in the case of switching to multipotency. Harvesting MSCs from bone marrow seems to be more appropriate in this case, as they are the easiest to collect; while collecting autologous neural stem cells from the CNS is possible, it can be troublesome and quite difficult, even if found within the olfactory bulb.^29^

It is also important to know the number of cells, that is, the dose, needed to achieve a therapeutic effect. As a rule, a minimal dose is in the order of tens of millions of cells.^30^ MSCs quickly become senescent, and differentiation efficiency is often very low.^31^, ^32^ The method by which cells are obtained for transplantation is related to their use in specific therapies for both non‐genetic and genetic diseases; hence, the pathophysiology of diseases and the changes they lead to may play a key role.

## 2 POTENTIAL USE OF STEM CELLS IN THE TREATMENT OF DISEASES WITHOUT A CLEAR GENETIC BASIS (NON‐HEREDITARY)

**FIGURE 2:**
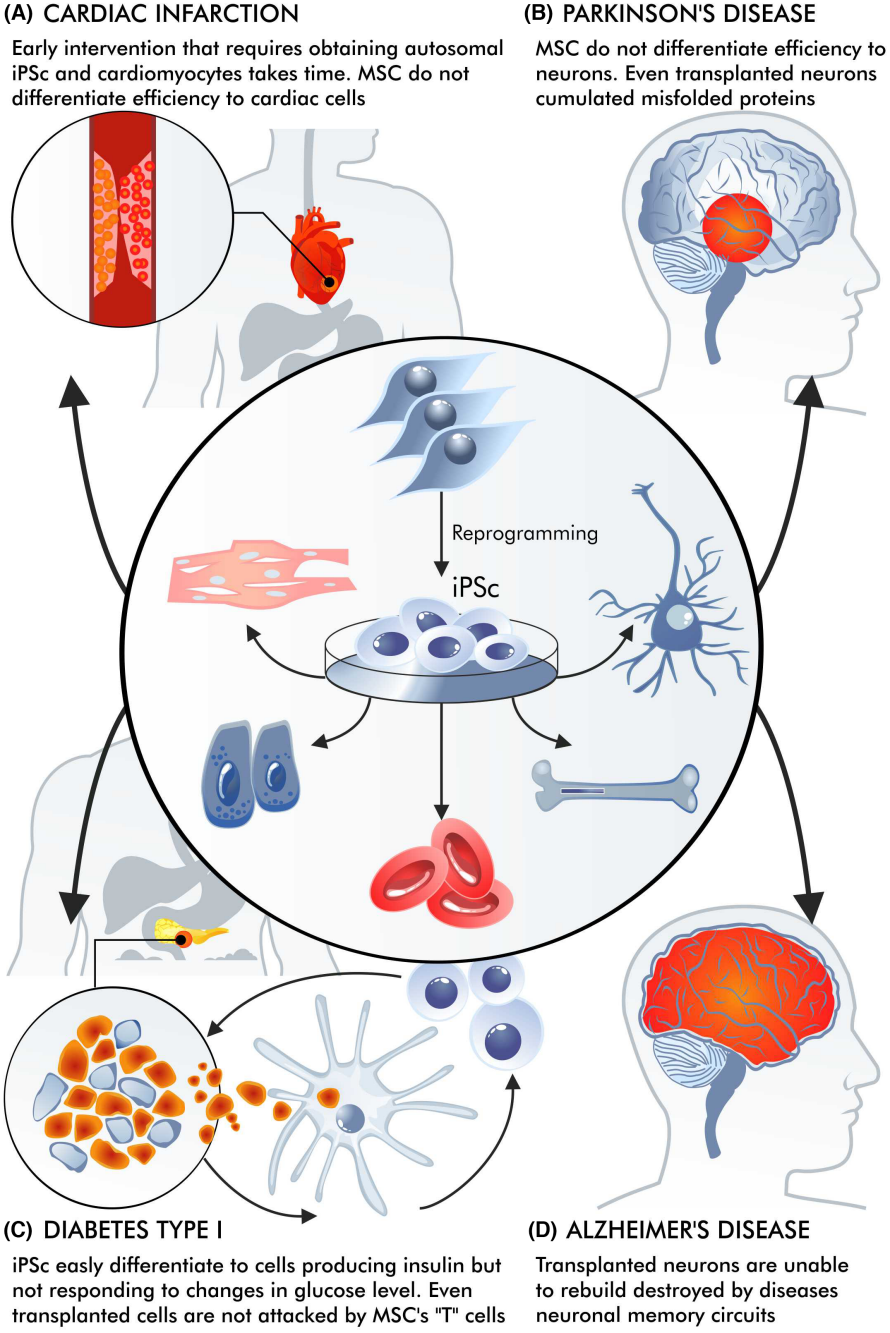
Examples of non‐hereditary diseases. Examples of non‐hereditary diseases were selected, representing myocardial infarction (A), Parkinson's disease (B), type I diabetes (C) and Alzheimer's disease (D). When trying to use cell therapy for sporadic diseases, various phenomena must be considered. In diabetes or even in Parkinson's disease, the original cause of the disease is not removed. Cell transplantation in diabetes type one does not stop T cell attacks on insulin‐producing cells. Misfolded proteins can be found in transplanted neurons (Parkinson disease). After a heart attack, scarring occurs very quickly, and the transplanted cells are unable to remove the scar and substitute it with new tissue. Alzheimer's disease causes massive changes in the CNS, and transplanted neurons are unable to rebuild the neuronal circuits that have been destroyed by the disease.

Cell therapies are used in a variety of situations.^33^ However, the requirements for using them vary considerably between a heart attack, a stroke, Parkinson's disease and type one diabetes (Figure 2). In the case of a stroke or a heart attack, a long wait for rehabilitation may prevent easy reversal of the effects of scarring^34^; it is also undesirable for the intervention to be too rapid, for example, immediately after an infarction.^35^ Contrary to some initial promises, cell therapy for diseases such as type one diabetes or Parkinson's disease does not eliminate the primary cause of the disease. It does not prevent the immune system from attacking the insulin‐producing cells and does not inhibit the accumulation of aggregates of misfolded proteins in neurons (Parkinson's disease).^36^

While obtaining cells that produce and secrete insulin in a glucose‐dependent manner is challenging, it is possible that those whose secretion is dependent on glucose can also alleviate the symptoms of type one diabetes. In addition, it has been found that therapy does not really work effectively for a long time. Transplanted cells can still be attacked by the cytotoxic lymphocytes that destroy insulin‐producing cells,^37^, ^38^ with the attack being hastened by the presence of allogeneic cells.^39^ Research is, therefore, aimed at protecting the cells from attacks by T lymphocytes,^38^ and the creation of insulin‐producing cells with edited genomes is being considered.^40^ However, progress is currently slow.

While stroke and heart attack do not involve a systematic attack by T cells, inflammation still plays a significant role in their pathology. However, it is important not to equate these conditions with autoimmune diseases like type 1 diabetes. Importantly, in the case of Parkinson's disease, where cellular therapies have been used more frequently, the aggregates of misfolded proteins can also be found in the transplanted neurons many years after transplantation.^41^, ^42^ This indicates that cell therapy alone cannot eliminate the primary molecular pathological mechanisms underlying these diseases.

In the case of a heart attack or stroke, intervention must be made relatively quickly before ‘scarring’ occurs.^43^ In this situation, MSCs could play a positive role. However, these may only be immunoregulatory activities. While their potential to differentiate into cardiomyocytes is interesting, their influence on immunoregulation or participation in the shaping of new vessels appears to be more important.^44^ MSCs offer less potential for differentiation into cardiomyocytes compared to iPSCs, that is, iPSCs differentiate to cardiomyocytes, whereas it is not obvious if MSCs differentiate or rather transdifferentiate to cardiomyocytes or cardiomyocyte‐like cells.^45^, ^46^, ^47^ While contracting cardiomyocytes were obtained after iPSC differentiation, no such phenomenon was found for MSC (trans)differentiation.

It is also difficult to use MSC in infarction therapy. Some authors suggest that the ability of MSCs to differentiate can be improved by genetic engineering.^48^ However, these procedures would also be time‐consuming, decreasing the chances of recovery after a cardiac infarction. MSCs and iPSCs vary significantly regarding their differentiation ability into cardiomyocytes. The reason why MSCs have a low efficiency in differentiating into cardiomyocytes may be due to low transcription of the appropriate genes, and combined with the rapid senescence of MSCs, the number of cells received, and their functionality are insufficient. Instead, a combination of these cell types is considered.^49^

MSC ‘differentiation’ can be supported by 5‐azacytidine (5‐aza).^50^ Antonitsis et al. also found that 5‐aza could stimulate bone marrow mesenchymal stromal cells (BMSCs) to differentiate into cardiomyocytes via random demethylation of DNA in the human body.^51^ Such compounds are not used during iPSC differentiation. This suggests that there may be epigenetic barriers hindering MSC differentiation into cardiomyocytes.^46^ However, it should be noted that 5‐azacytidine (5‐aza) is cancerogenic.^52^

While the pathways of iPSC differentiation are relatively well defined, it is very difficult to find similar data referring to MSC differentiation. As such, few molecular explanations exist for differences in iPSC versus MSC cardiomyocyte differentiation potential.^53^ A similar situation will occur if someone applies MSC after a stroke. iPSC cultures can provide any number of mature cells.^54^ However, given the long iPSC preparation procedures, this may not be adequate when timely intervention is crucial for patients in need of treatment. Therapy after this time may bring some improvement, but it will not be fully effective. The production of universal cells, that is, cells with certain HLA‐edited genes that can be taken by any recipient, may have potential for treating patients after a heart attack or a stroke. The most common targets in this area include knockouts of the beta‐2‐microglobulin gene.^55^ For these reasons, allogeneic transplants are also being considered after taking cells from deceased donors, but such an approach does not seem to be effective. On the contrary, allogeneic MSCs have been used to great effect in treating patients with spina bifida, as proposed by Farmer.^56^ The authors provided placenta‐derived MSCs localized on a specific scaffold at the early stage of fetal development. Closing the bifida and stopping the exposure of the spinal cord to amniotic fluid at this stage resulted in normal fetal development. In these studies, MSCs were found to be better than iPSCs. These findings also highlight the importance of early therapy; however, this approach will not be successful if a child has already been born with spina bifida. As with spina bifida cell therapy, other effects may depend on the use of biopolymers.^57^

Other problems are encountered in the case of neurodegenerative diseases, although this section does not deal with family cases of Alzheimer's or Parkinson's disease. In sporadic Parkinson's disease, administration of dopaminergic neurons to a damaged substantia nigra can result in some improvement.^58^ These patients may wait longer for neuronal cells. For safety reasons, work on allogeneic transplants continues on MSCs rather than iPSCs; however, there is a significant risk of transplant rejection in this case.^59^ In Alzheimer's disease, the situation is much more complicated because it is impossible to identify a specifically localized place where neuronal cells can be administered. For example, the loss of a specific memory (remembrance) results from damage to a certain neuronal circuit or its loss. Additionally, it is also more difficult to obtain an accurate model for animal studies.^60^ Memory neuronal circuits that have been lost due to neurodegeneration can be reconstructed by transplanted stem cells or neurons. However, therapeutic progress is likely to be slow.^61^ Lately, glial cell transplants have been proposed as a new approach to treatment, but they have only been used so far in animal models. Replacing glial cells can be more effective since their supportive role is important and those cells do not form circuits.^62^

As for MSCs, they seem to have certain potential for use in connective tissue diseases, for example, osteoarthritis.^63^ However, the situation here is also complicated. If the disease is in its early stages, weight loss and engaging in physical activities will have greater effects. If the disease is advanced, prosthetics work significantly better than cell therapies. Allogeneic bone marrow cell transplants have been used for decades and are an excellent example of successful transplantation when the transplanted cells do not need to form complex 3D structures. When more complicated structures are required, derived iPSCs are sometimes more plausible than allogeneic MSC bone marrow transplants.^64^ Still, diseases related to soft tissues are more susceptible to MSC activity and differentiation. Osteoarthritis serves as a prime example of such a condition. MSCs show important immunoregulatory and anti‐inflammatory properties, in addition to the ability to differentiate into cartilage cells. Clinical trials have demonstrated that MSC‐based therapy can have positive effects in osteoarthritis patients.^63^

On the whole, MSCs have been tested for neurological diseases. Many well‐designed clinical trials have been conducted for patients with stroke. Zheng et al. showed that the treatments were safe for stroke patients, and some minor improvements were observed.^65^ Moreover, autologous MSC treatment led to an improvement in the status of patients with burns, with an increased cure rate and shorter hospitalization.^66^

Many cell therapies involve very careful consideration of which cell types should be given. An example is vision restoration, where it is possible to transplant retinal stem cells, or rods and cones.^67^ However, stem cells do not have to differentiate into neurons (rods and cones) and can differentiate into glia; also, additional treatments may be needed to ensure that the rods and cones perform their function after transplantation.^68^ As indicated by these examples, cell therapy is highly complex; it requires time to prepare the cells, whereas the pathophysiology of the disease is not well understood.

## 3 CELL THERAPY IN HEREDITARY DISEASES

**FIGURE 3:**
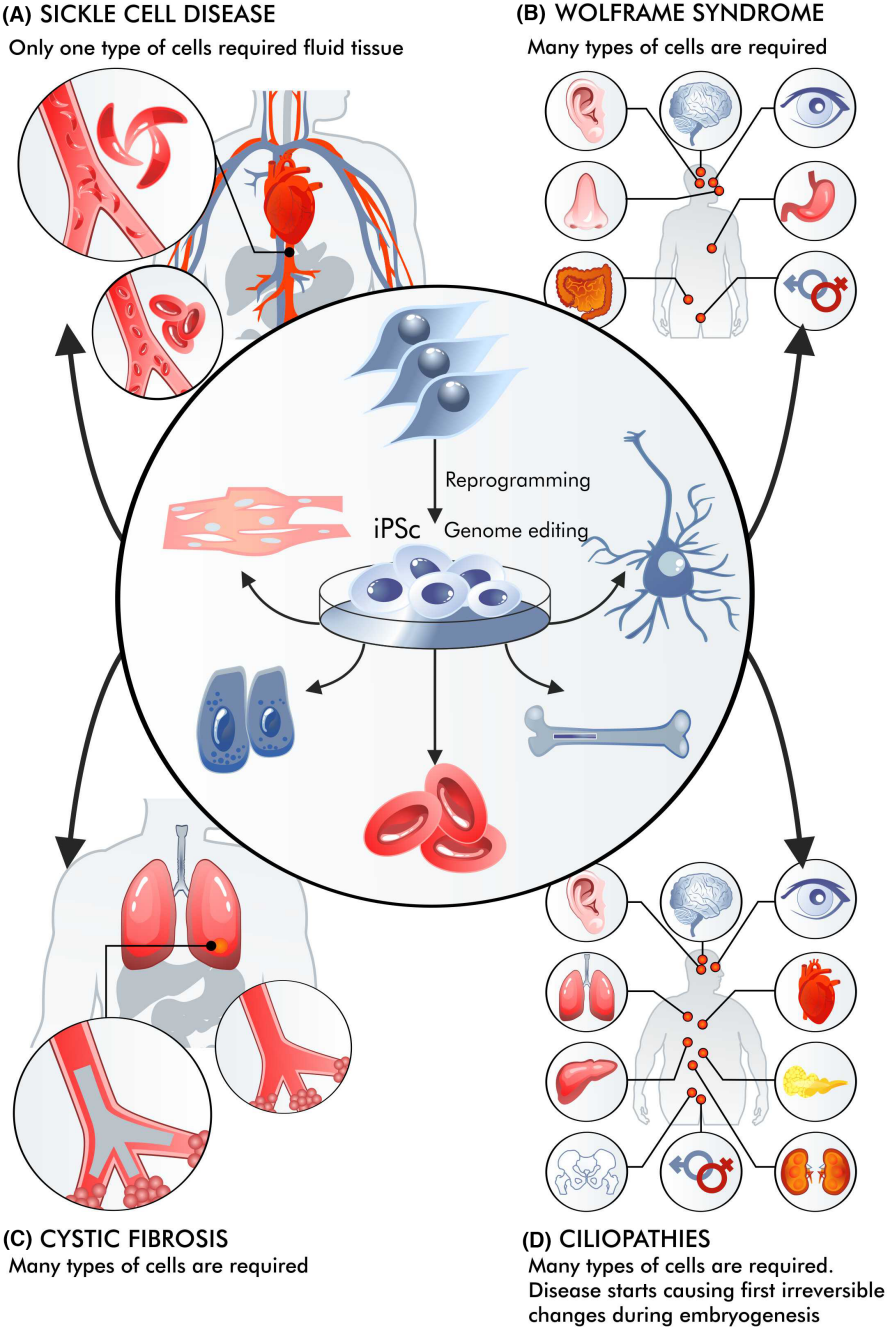
Problems of cell therapy in the example of selected genetic diseases (disorders). Patients with different genetic diseases can be treated with varying degrees of success. In anaemia (A), the chances are great because the disease affects one type of cell that can be corrected, and their stem cells are relatively easily delivered. On the contrary, other diseases affect many types of cells, making them extremely difficult to treat: Wolfram's syndrome several types of stem cells and mature cells are becoming apoptotic (B), cystic fibrosis, and all cells showing cilia are targeted by the disease from the respiratory system through the digestive system and fertilization glands. (C) as well as ciliopathies (D). Almost all cells depend on single cilia for proper action. These diseases start at the earliest stages of human disease, and malfunctions can be irreversible.

With hereditary diseases, the situation is even more complex than with diseases without a clear hereditary (genetic) basis (Figure 3). For example, sickle cell disease differs from haemophilia or cystic fibrosis, and an even more complicated situation can be observed in Wolfram syndrome or ciliopathies.^69^, ^70^, ^71^ The use of cell therapy can be viewed as part of gene therapy since stem cells can be genetically modified. In such cases, iPSCs are desirable, as under suitable conditions, they can proliferate without limits. HSCs (haematopoietic stem cells) can be obtained for the patient and modified in vitro, making it possible to avoid in vivo administration of high doses of vectors, which can be particularly dangerous for chronically ill patients. This was particularly evident in the case of cystic fibrosis.^72^

Currently, for some mutations, higher expectations are placed on genome editing than on the introduction of therapeutic transgenes.^73^ Genome editing methods are developing rapidly, from ZFN through CRISPR to PE.^74^ Although not all editing methods are equally easy to execute, there is a huge difference between knocking out genes and correcting their sequence. There are also many problems with the specificity of these methods, such as editing the genome outside the predetermined location/gene.^75^

Such therapy can play a positive role in sickle cell disease. iPS cells can be obtained, edited and differentiated into haematopoietic stem cells (HSCs). If the patient is given autologous HSCs with the correct Hb gene (optimally after editing), these cells can differentiate into erythroblasts and yield long‐term improvement. It is also possible to edit HSC genomes to increase the number of their divisions.^76^ In the case of iPSCs, there are even attempts to create libraries for recipients with different HLAs.^77^ A similar situation occurs in beta‐thalassemia.^78^ iPS cells have a relatively short half‐life, and patient status may gradually improve after multiple transplants.

In genetic disorders like Wolfram syndrome, the accumulation of misfolded Wolframin protein in miscellaneous cells damages various stem cells across different cell types.^79^ The patient develops symptoms of diabetes, a gradual onset of deafness, and blindness as ER stress is involved. The misfolding of the Wolframin protein also results in the death of many stem cells, exacerbating the condition. Moreover, it is not possible to administer so many types of stem cells, which would solve the problem. Such diseases are more difficult to treat than those resulting from mutations of genes expressed only in a selected group of cells or in erythroblasts or reticulocytes. In this case, iPSCs are more used to create disease research models than therapies.^80^

In other cases, pathophysiological changes may begin in the earliest stages of life, leading to irreversible malfunctions. Ciliopathy is a good example of a disease in which a mutated gene is expressed in almost all cells.^81^ The first pathological changes can already be observed during embryogenesis.^82^ As the organism develops and matures, these changes are both progressive and complex. It is then difficult to imagine that they can be reversed after stem cell transplantation.

The next aspect that is often ignored is the immunological response, namely that after the introduction of a transgene or after editing, the cells are not completely autologous; they are accompanied by a protein that may be new to the donor's immune system.^83^, ^84^ However, in conditions such as sickle cell disease, a change in an amino acid is unlikely to trigger a humoral or cellular response against cells presenting this new protein. Very often, however, the disease may be recessive, with the protein not present due to the deletion of an entire gene or by a mutation that causes the STOP codon to appear earlier, leading to nonsense mRNA decay.^85^ If the protein was absent before genome editing, its introduction causes the APC to present the appropriate peptide (epitopes) using MHC molecules.^83^, ^84^ It is quite difficult to predict the consequences of such a phenomenon, but it is likely to resemble the appearance of the immune system response after the administration of factor VIII to a patient with haemophilia A.^86^ More generally, the presence of transgenes of immunogenic proteins involved in genome editing, such as CAS9, may also cause a problem; hence, the possibility of switching off transgene expression should be pursued.^87^

The diverse opportunities associated with cellular therapies for hereditary diseases are accompanied by specific obstacles; however, these are gradually being overcome.

## 4 HOPES RELATED TO SYNTHETIC BIOLOGY

Synthetic biology enables the design of new cell types and the modification of existing ones. A classic example of cell formation as part of synthetic biology is the chimeric antigen receptor T cell (CAR‐T) design.^88^ In the basic version, these modified cells eliminate cancer cells. The CAR‐T is created by inserting a transgene encoding a synthetic CAR into lymphocytes. Autologous CAR‐T therapies are quite successful in treating patients with certain leukaemias and lymphomas, such as B‐cell acute lymphoblastic leukaemia and refractory diffuse large B‐cell lymphoma.^89^ However, autologous CAR‐T or CAR‐M (M‐macrophages) cells must be prepared for individual patients, and not all of them survive until the therapeutic cells are prepared. The isolated T cells are often damaged by disease and chemotherapy and can cause side effects such as a cytokine storm. These issues can be solved by treatment with a universal but genetically engineered CAR‐T; most importantly, the patient will not need to wait for CAR‐T transplantation. The source of the cells is independent and remains intact through therapy and disease. New types of synthetic receptors may provide the opportunity to minimize the risk of a cytokine storm or CAR‐T exhaustion.^90^ As such, it may be possible to create many types of artificial or synthetic cells with synthetic receptors.^91^

**FIGURE 4:**
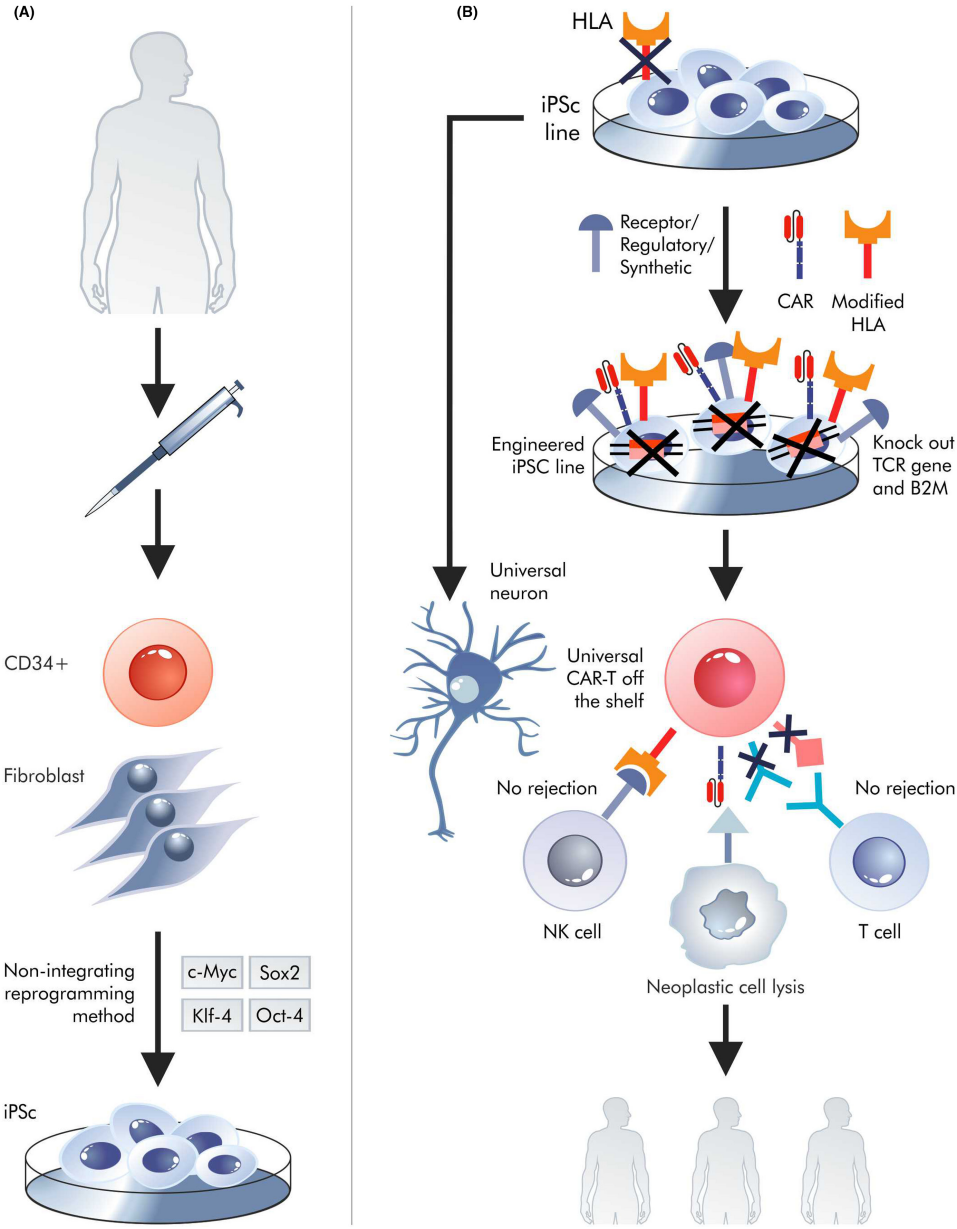
Universal cells. Obtaining universal cells for each recipient also opens the way for the creation of subsequent editions of the genome and the introduction of transgenes enabling a certain autonomy, for example, CAR‐T cells. It may consist of an adequate response to environmental changes such as a cytokine storm, the escape of cancer cells from a CAR‐T attack or the on‐target off cancer phenomenon. To make this attempt possible, we can perform reprogramming of HSC and/or fibroblasts into iPSC (A), which can then be genetically modified by the knockout of genes involved in immunological response (TCR and B2M). Hence, these cells can be implemented for any patient as a universal CAR‐T off‐the‐shelf product (B).

Another important thing is the creation of universal cells for each recipient, that is, *off*‐*the*‐*shelf*.^92^ This may mean the formation of universal cells with synthetic receptors or, more broadly, synthetic proteins acting within universal cells (Figure 4). Universal cells do not need to be CAR‐T; in fact, other universal cell types can be more easily deployed. Obtaining universal cells requires advanced editing of the genome within the human leukocyte antigen (HLA) genes involved in rejection of transplant and growth versus host. However, the greatest difficulty is associated with CAR‐T cells, as they can be attacked by the recipient's immune system and also attack healthy acceptor (host) cells using their own TCRs. As a rule, they attack antigen‐presenting cells or epitopes unidentified during T cell thymus selection; however, even peptide‐free MHC‐I molecules can be incorrectly recognized as targets. It is easier to get one genetic (HLA) modification required to obtain universal cardiomyocytes than two (HLA and TCR) needed for universal CAR‐T. However, it does not mean that this approach would be trivial. Simple removal of MHC can induce an attack by NK lymphocytes.^93^ In addition, necrosis of universal cells always leads to the release of their non‐autologous antigens. Of course, CAR‐M does not express T cell receptor (TCR) genes; however, their cytotoxic potential against cancer cells is not fully understood. At the iPS cell level, techniques such as CRISPR or PE can be used to obtain neurons, cardiomyocytes or CAR‐T cells.^94^, ^95^

Among other things, synthetic biology allows the transgene expression needed for genome editing to be turned off. Although in this case, episomal vectors can be considered when creating iPSCs.^15^ It should be mentioned that the term ‘universal CAR‐T’ sometimes implies the ability to obtain cells containing chimeric antigen receptors that switch scFv from recognizing one antigen to another.^93^ In this case, the term refers to cells created for particular recipients. Similarly to non‐genetic diseases, universal cardiomyocytes could solve some problems in the event of a heart attack because they could be administered to the patient before the scar forms. The possibilities for genetic modification of iPS universal cells are theoretically extensive. However, there are many complications. For example, due to the short half‐lives of CAR‐T, it is not possible to modify many genes or introduce multiple transgenes encoding synthetic receptors into the donor T cells in vitro; however, it can be done at the iPSC level.^96^ CAR‐T technology shows many problems that can be solved through modifications done during universal cell generation, such as cytokine storms,^97^ exhaustion^98^ and the *on target off cancer* phenomenon.^99^ All these problems can be solved by introducing appropriate transgenes, including those for synthetic receptors, into iPSCs or by editing their genome to allow the production of synthetic types of T lymphocytes. These actions, which increasingly resemble the formation of integrated circuits in electronics^100^ (Figure 4), will work inside synthetic universal cells.

**FIGURE 5:**
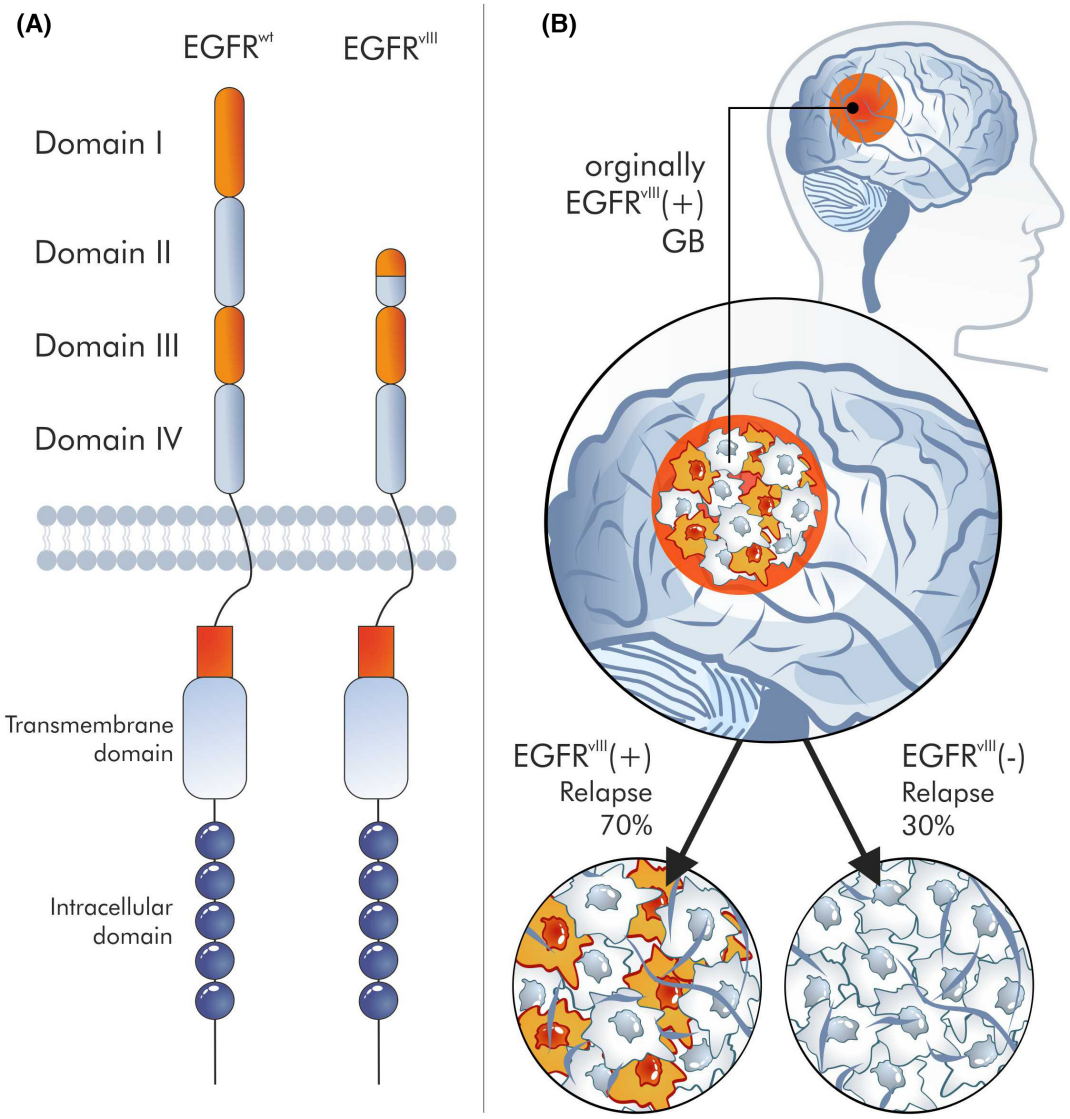
EGFRvIII as an example of a target for immunotherapy. EGFRvIII, a deletion mutant of EGFRwt, is an example of a target for immunotherapy that seemed ideal, but the immunotherapies targeting it are disappointing. It is a membrane antigen, and it is characteristic of cancer cells (A). However, the role of this protein is enigmatic. It is uncertain if glioblastoma cells are dependent on it or not. Unfortunately, it is only present on some cancer cells and does not currently appear to be a CSC marker. In addition, it is observed that the recurrence does not show its expression, even though it was present in the primary lesion (B).

However, the operation, function and role of targets for CAR‐T or other CARs, for example, CAR‐M, remain unclear. A classic example would be EGFRvIII,^101^ which seems to be a great target for CAR,^102^ because it is only found in cancer cells. EGFRvIII is an EGFR mutant with an 801 bp deletion of the region encoding the N‐terminal domains. Hence, the mutated receptor is no longer able to bind ligands but can undergo covalent and noncovalent dimerization in the absence of a ligand. Most likely, the deletion of the receptor part is a consequence of recombination between Alu sequences, flanking the junction sites of introns 1 and 7 of the EGFR gene. EGFRvIII is only present in a certain percentage of tumour cells.^103^ These fractions of EGFRvIII‐positive cells are generally marginal, although these cells are present in 30% of glioblastoma (GB) cells.^103^ It is unknown what will happen to the remaining 70% of EGFRvIII‐negative cells after the elimination of these 30% of EGFRvIII‐positive cells. Studies refute the hypothesis that EGFRvIII is a marker for tumour stem cells^104^ and therefore that their elimination will result in the elimination of the entire tumour; indeed, this antigen is absent in some relapses (Figure 5). Also, immunotherapies had little success against EGFRvIII‐positive cells.^105^ Clearly, cell therapy is a complex procedure with many dependent variables, and although synthetic biology opens new perspectives in cell therapy, this is accompanied by many problems that must be solved.

## 5 ARE SYNTHETIC EMBRYOS, OR SHEEFS, A POSSIBLE SOURCE OF ELEMENTS FOR TRANSPLANTATION?

One of the problems of regenerative medicine is the creation of complex 3D structures. On the verge of cell therapy lie synthetic embryos and SHEEF structures.^106^ ESCs have been found to self‐assemble in vitro into 3D structures known as assembloids, gastruloids, blastoids and organoids,^107^, ^108^ and SHEEF analyses have been carried out for years. Mouse ESCs combined with eTSCs (trophoblast stem cells) self‐organize into blastoids with a blastocyst‐like structure.^109^ Baccari et al. describe gastruloid aggregates made of mouse ESCs^110^; however, these 3D structures are unable to form embryos after being transferred to the uterus^109^ and cannot develop into more advanced stages of embryogenesis.^111^

**FIGURE 6:**
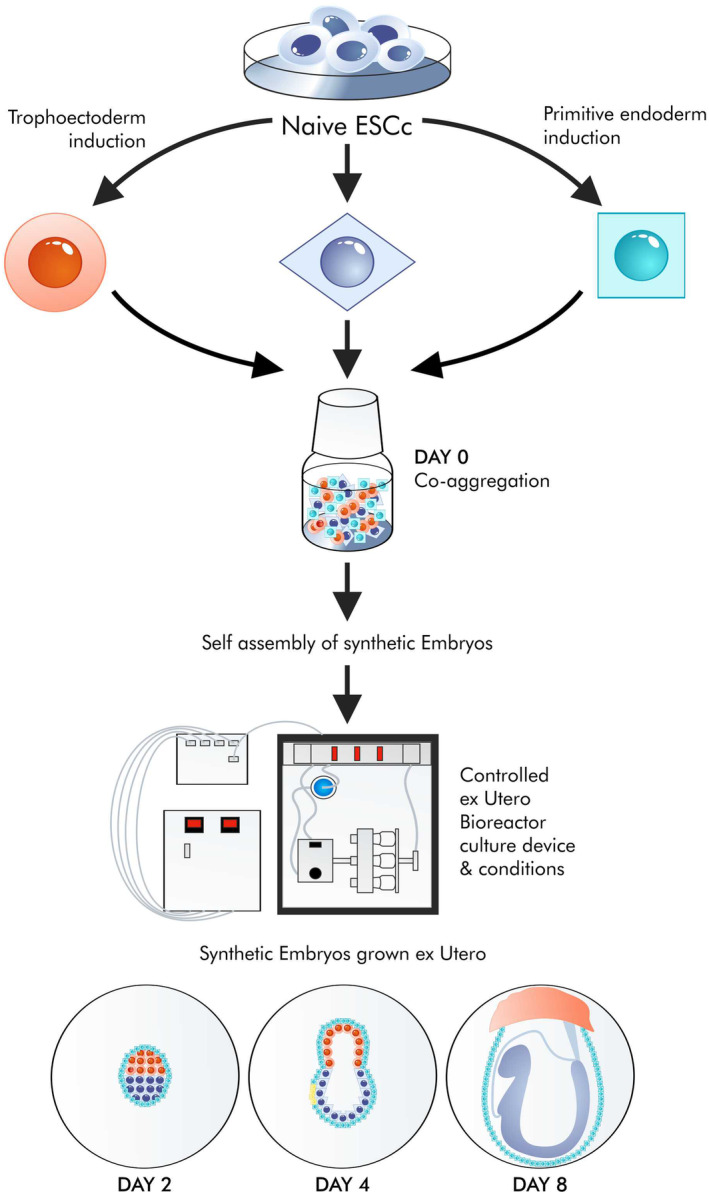
Synthetic embryos. The method of obtaining synthetic embryos is based on the self‐organization of appropriate cells, for example, pluripotent cells or trophoectoderms grown in a bioreactor, and controlling many parameters such as oxygen partial pressure or glucose concentration. In these conditions, so‐called synthetic embryos are formed, which, according to some creators of the method, may be the source of cells for transplantation. This is an approach that does not comply with current legislation and does not solve many transplant problems or give an advantage over iPSC differentiation. For example, insulin‐producing cells are still going to be like fetal cells, not responding to changes in glucose levels.

Two key papers were recognized by developmental scientists as a major breakthrough.^112^, ^113^ They suggested that synthetic embryos could be sources of cells and tissues ‘as their 3D printers’. Those organisms self‐organize from cells supplied to the bioreactor^112^ (Figure 6). While the concept itself is shocking from an ethical perspective regarding human embryos, it is also technically quite controversial. The formation of synthetic embryos, or SHEEFS, is related to the arrangement of stem cells, for example, trophoblast cells, into more advanced 3D structures. In bioreactors outside the uterus, synthetic embryos reach a state in which the beating heart, the CNS elements and the gastrointestinal tract emerge.^113^ They cannot remain viable in the bioreactor at this stage of development, and it is obviously no longer possible to implant them into the uterus.

In contrast, iPS cells do not allow for the formation of organized organs but rather chaotic teratomas. To this end, research is aimed at determining whether an intermediate state can be achieved that is ethically acceptable or justifiable.^114^ Such a state is the SHEEF, which is capable of creating complex structures containing organs but without the status of an organism.^115^

It should be emphasized that agreeing to the production of synthetic embryos will not solve most of the previously described pathophysiological problems. Firstly, as noted above, even cells producing insulin only in response to changes in glucose levels will not stop T cells from attacking. Also, fetal cells are unable to react to changes in glucose concentration as adult cells can. In addition, the creation of human GMOs is illegal.^116^ Controversial projects such as synthetic embryos are therefore not legally applicable to activities such as receiving modified cells for people with genetic diseases. Many legal regulations, such as the rulings of the Court of Justice of the European Union, also prohibit the patenting of such technologies.^117^ Even if the creation of synthetic embryos for the purposes of 3D structure printing is allowed (which is doubtful), there is no possibility of obtaining insulin‐producing cells other than those resembling fetal insulin‐producing cells, that is, fully mature cells that produce insulin and, most importantly, secrete insulin depending on glucose concentration.

Similar problems occur when it comes to the differences between fetal cells and adult cells. Hence, it seems that iPSC and MSC‐based synthetic biology offer solutions that are more practical, ethical and achievable from a rational perspective.^118^, ^119^, ^120^

## 6 CONCLUSION

Although cell therapies offer considerable hope as a method of treatment, it is important to be mindful of the unique requirements of their targets. The pathophysiology of disease often involves irreversible scarring and traumatic changes, and after stroke or infarction, transplantation requires perfect timing to succeed. Early intervention can lead to worsening symptoms, and late intervention might decrease the chances of regeneration due to scarring. In such situations, universal cells, that is, for any recipient, can be a game changer as they allow the optimal time of implantation to be adjusted. Unfortunately, cell therapies do not remove the primary cause of diseases such as type one diabetes or even Parkinson's disease. It means that cell therapies for diseases should be combined with a treatment that removes the primary pathologic factor, such as the destruction of transplanted pancreatic cells in type one diabetes. In the case of hereditary diseases, it is necessary to distinguish between conditions with different treatment requirements, such as sickle cell disease, haemophilia and Wolfram syndrome.

Many complications exist regarding mutations, cell type and the impact of pathological changes that are resolved throughout life. Sickle cell diseases can be treated more easily than Wolfram syndrome, with many cell types located in many organs and malfunctioning.

While the use of synthetic embryos has many ethical problems, synthetic biology offers promise due to the universality or autonomy of the emerging cells. MSCs have been found to have promise in spina bifida, osteoarthritis and various neurological diseases. Autologous MSCs have resulted in improvements in patients with skin burns.

CAR‐T therapy offers hope for the future of cellular therapies. Combined with genome editing and synthetic biology, new cell types can help treat many patients with cancer (neoplasia). So far, CAR‐T therapies are effective and relatively safe treatments for patients with haematological malignancies.

Although new solutions with different types of synthetic receptors and synthetic cells are under development, it will probably be many years before they are approved for use. Progress in cell therapy is laborious, and many problems must still be identified and overcome.

## AUTHOR CONTRIBUTIONS

**Cezary Tręda:** Formal analysis (equal); writing – original draft (equal); writing – review and editing (equal). **Aneta Włodarczyk:** Formal analysis (equal); writing – original draft (equal); writing – review and editing (equal). **Piotr Rieske:** Conceptualization (lead); formal analysis (equal); funding acquisition (lead); project administration (lead); supervision (lead); writing – original draft (equal); writing – review and editing (equal).

## FUNDING INFORMATION

Polish Chimeric Antigen Receptor T‐cell Network, Car‐NET, 2020/ABM/04/00002‐00.

## CONFLICT OF INTEREST STATEMENT

The authors declare no conflicts of interest.

## Data Availability

Data sharing not applicable to this article as no datasets were generated or analysed during the current study.
